# Uncovering the Effect of Al Addition on the Hydrogen Storage Properties of the Ternary TiVNb Alloy

**DOI:** 10.3390/ma15227974

**Published:** 2022-11-11

**Authors:** Nayely Pineda-Romero, Claudia Zlotea

**Affiliations:** Univ. Paris Est Creteil, CNRS, ICMPE, UMR 7182, 2 rue Henri Dunant, 94320 Thiais, France

**Keywords:** multi-principal element alloys, hydrogen storage, thermodynamics, synchrotron X-ray powder diffraction

## Abstract

The effect of Al addition on the structure, microstructure and hydrogen storage properties of the ternary TiVNb alloy was investigated from small amounts to equimolar composition. Al_x_(TiVNb)_1−x_ (x = 0.05, 0.175 and 0.25) alloys are *bcc* single-phase materials with decreasing lattice parameters with increasing Al content. Al addition progressively decreases the hydrogen storage capacity but also destabilizes *fcc* dihydride formation for alloys with x ≤ 0.10. Among the different compositions, the most promising alloy was found to be that with x = 0.05 Al content that exhibited high initial storage capacity (2.96 wt.%), a less stable hydride (ΔH = −52 kJ/mol H_2_ and ΔS = −141 J/K∙mol H_2_), better desorption properties (desorption onset temperature around 100 °C) and enhanced reversible capacity during cycling (2.83 wt.%) compared to the ternary TiVNb. In situ and ex situ synchrotron X-ray powder diffraction, together with thermal desorption experiments, showed improved desorption properties with Al addition, together with a two-step reaction with hydrogen. These findings highlight the use of small quantities of lightweight Al in refractory multi-principal element alloys as a promising approach for enhancing the solid-state hydrogen storage performance of *bcc*-type alloys.

## 1. Introduction

In the context of climate change, hydrogen represents an opportunity to mitigate global warming as it is a zero-carbon fuel with three times more energy per mass than fossil fuels (142 MJ/kg). However, its energy per volume is very low and its storage necessitates close attention to ensure that it is a safe, affordable, and efficient technology. Available storage technologies include compressed gas and liquid hydrogen. In addition, metal hydrides used for solid-state hydrogen storage appear to represent a positive storage option that can meet required criteria. However, they face several challenges including sluggish absorption/desorption kinetics, unfavorable thermodynamics and fading reversible capacity [1,2]. Nevertheless, metal hydrides represent one of the safest options for stationary applications and show high volumetric hydrogen storage capacity in the range 60–100 Kg/m^3^, which is higher than that of compressed gas or liquid hydrogen. These properties make them good candidates for the investment of research effort to overcome their limitations and achieve efficient hydrogen storage systems which can operate in near ambient conditions [3].

A new concept in metallurgy was established in the work of Cantor et al. [4] and Yeh et al. [5] by the introduction of multi-principal element alloys (MPEAs). MPEAs have significant atom fractions of several elements in which the distinction between minor and major elements vanishes. Four or more elements at close to equimolar concentration are commonly used in MPEAs. Alloys with five or more elements are called high entropy alloys (HEAs). The main motivation for increasing the number of alloying elements is to maximize the configurational entropy to improve the stability of disordered solid solution phases, thus suppressing the formation of intermetallics [1,3]. MPEAs often form random solid solutions, adopting simple crystalline lattices, such as face-centered cubic (*fcc*), body-centered cubic (*bcc*) or hexagonal compact (*hcp*) structures. The large lattice distortion (δ) which may be created by sharing the same crystalline sites among many atomic constituents is a key factor that can create large interstitial sites to accommodate hydrogen. Among recent attempts to use MPEAs for hydrogen storage, *bcc* alloys have been proposed as good candidates to form metal hydrides [3]. The equimolar TiVZrNbHf alloy reported by Sahlberg et al. crystallizes in a *bcc* single phase and absorbs hydrogen within a single-step reaction forming a body-centered tetragonal (*bct*) hydride with a high capacity of 2.5 hydrogen atoms per metal atom (H/M) [6]. Later, Zlotea et al. described equimolar TiZrNbHfTa HEA, which also adopted a *bcc* lattice with a maximum capacity of 2.0 H/M [7]. The hydrogen absorption pathway is based on a two-step reaction: first, the alloy absorbs hydrogen at low pressure, transforming into a monohydride phase (capacity∼1.0 H/M), followed by a second transition at high pressure from the monohydride to a dihydride phase (capacity~2.0 H/M). Most reported HEAs follow this two-step reaction with variable limits for monohydride and dihydride capacities. 

Non-equimolar compositions have also recently attracted attention, such as the *bcc* Ti_0.325_V_0.275_Zr_0.125_Nb_0.275_ alloy that showed a maximum capacity of 1.7 H/M [8]. Starting from this composition, the same authors demonstrated an increase in the total capacity up to 2.0 H/M by adding 10 atomic percent (at.%) of Ta, Cr or Mo (Ti_0.30_V_0.25_Zr_0.10_Nb_0.25_*X*_0.10_ *X* = Ta [9], Cr [10] and Mo [11]), without affecting the *bcc* lattice of the initial solid solution phase. Despite these improvements, these alloys possess modest gravimetric capacities because of the use of quite heavy elements. Therefore, an important feature to consider in the field of HEAs for hydrogen storage is the increase in gravimetric capacity that can result from the introduction of lightweight elements [1]. However, only a limited number of reports have addressed the use of lightweight *bcc* HEAs for hydrogen storage purposes. The addition of low-density metals (Mg, Al, or Sc) to increase the hydrogen gravimetric capacity in HEAs involves complex interactions with the alloying elements, which has sometimes resulted in missing the target of enhancing capacity [1,3]. Strozi et al. investigated the *bcc* MgVAlCrNi alloy containing two lightweight metals (Mg and Al) but it exhibited a low hydrogen capacity (0.30 wt.%) that was attributed to the positive enthalpy of the hydrogen solution in most of the alloying elements, including Mg [12]. The MgAlTiFeNi alloy reported by Cardoso et al. crystallized in a *bcc* lattice and also exhibited limited H_2_ capacity (0.94 wt.%) [13]. Ferraz et al. investigated the Mg_35_Al_15_Ti_25_V_10_Zn_15_ alloy with the objective of increasing the gravimetric capacity by use of both Mg and Al. However, the material synthesized by reactive ball milling under an H_2_ atmosphere was multiphase, composed of an *fcc* hydride together with MgH_2_, with a total capacity of 2.75 weight percent (wt.%) [14]. Finally, the material behaved as a simple mixture of several hydrides. Montero et al. produced interesting results by the addition of Mg or Al to the Ti_0.325_V_0.275_Zr_0.125_Nb_0.275_ alloy. The Mg_0.10_Ti_0.30_V_0.25_Zr_0.10_Nb_0.25_ and Al_0.10_Ti_0.30_V_0.25_Zr_0.10_Nb_0.25_ alloys occurred as *bcc* single phases with a maximum capacity of 1.7 H/M (2.7 wt.%) [15] and 1.6 H/M (2.6 wt.%), respectively [16]. Furthermore, in our previous investigations, we demonstrated that the addition of 10 at.% of Al into the ternary *bcc* TiVNb alloy decreased the storage capacity from 2.0 H/M (3.2 wt.% for TiVNb) to 1.59 H/M (2.6 wt.% for Al_0.10_(TiVNb)_0.90_) [17]. Despite the observed decrease in capacity, other aspects of performance were positively affected by the presence of Al. Al addition (i) destabilized dihydride formation (from the monohydride to the dihydride phase), (ii) flattened the plateau pressure, which is a required condition for the practical use of metal hydrides, (iii) decreased the desorption onset temperature below 100 °C, and (iv) increased the stable capacity over absorption/desorption cycling.

Consequently, further research is needed to increase knowledge of the hydrogen storage properties of lightweight HEAs with a view to achieving high hydrogen capacity, fast hydrogen absorption/desorption kinetics close to standard temperature and pressure (STP) conditions, favorable thermodynamic properties, and high reversible hydrogen capacity during cycling [3]. Based on the above, we report on the hydrogen sorption properties of the lightweight series of alloys, Al_x_(TiVNb)_1−x_ with x = 0.05, 0.175 and 0.25, synthetized by arc melting. Our objective was to vary the Al content to tune the microstructure, physicochemical, and hydrogen sorption properties. For this purpose, laboratory and large-scale experimental facilities were combined to clarify the effect of the amount of Al introduced into TiVNb. Well-known laboratory techniques, as well as ex situ and in situ synchrotron X-ray powder diffraction, were carried out and the results compared to our previous results for ternary TiVNb and quaternary Al_0.10_(TiVNb)_0.90_ alloys [17]. The thermodynamic experimental results obtained were compared with data-driven predictions facilitated by a previously published machine/statistical learning (ML) model [17]. We sought to perform a comprehensive analysis by varying the Al content introduced into TiVNb from low amounts to equimolar composition.

## 2. Materials and Methods

The Al_x_(TiVNb)_1−x_ (x = 0.05, 0.175, 0.25) compositions were synthetized using an electric arc melting method from bulk pieces of Ti (99.99% pure, Alfa Aesar), V (99.9% pure, NEYCO Vacuum&Materials), Nb (99.95% pure, Alfa Aesar) and Al (99% pure, STREM Chem). Due to the high melting points of Ti, V and Nb (2468 °C for Nb > 1902 °C for V > 1660 °C for Ti) these three metals were pre-alloyed in an electric arc furnace under Ar atmosphere (around 300 mbar) and remelted 16 times by flipping the ingot between each melting to achieve good homogeneity. Due to its low melting point (660 °C) and to avoid loss of material, Al was added to the pre-alloyed TiVNb and melted under the same conditions as above with eight repetitions. The mass loss during the synthesis was less than 1% over the total mass of alloy (3 g).

The hydrogenation process was carried out using a Sievert apparatus, which consisted of a home-made manual manometric device with thermostatically calibrated volumes. A stainless-steel sample holder was filled with about 400–500 mg of sample cut into small pieces, sealed using metal gaskets to prevent gas leakage and connected to the Sievert apparatus. Before starting the hydrogenation process, an activation procedure was carried out by exposing the sample at 410 °C under dynamic vacuum (~1 × 10^−5^ mbar) overnight. Two types of measurements were carried out: absorption kinetics at 25 °C and pressure-composition isotherms (PCIs) for a range of temperatures. For the absorption kinetics, the sample holder was placed in a water bath under isothermal conditions (25 °C) and a final equilibrium pressure (P_eq_) of around 38 ± 5 bar of H_2_. For acquisition of the PCIs, small doses of hydrogen were used up to around 47 bar. A resistive furnace was used for the acquisition of PCIs at high temperatures (above 25 °C). The mass of the samples was measured carefully before hydrogenation to calculate the storage capacity using the real gas state equation for H_2_ in GASPAK Version 3.32 (Horizon Technologies). For the absorption/desorption cycling experiments, absorption was performed at 25 °C and a final P_eq_ around 32 ± 4 bar H_2_, while desorption was performed by heating at 410 °C under secondary vacuum (~1 × 10^−5^ mbar) for 12 h.

The crystalline structure of the samples was investigated by laboratory X-ray powder diffraction (XRD) using a D8 Advance Bruker diffractometer (Cu K_α_ radiation λ = 1.5406 Å, Bragg-Brentano Geometry). In addition, both ex situ and in situ structural characterizations were carried out by synchrotron X-ray powder diffraction (SR-XRD) at the Cristal beamline of the SOLEIL facility. The intermediate hydrides of TiVNb and Al_0.05_(TiVNb)_0.95_ compositions were placed into 0.2 mm diameter borosilicate capillaries and characterized by ex situ SR-XRD in the scanning range 10° to 60° (2θ) with the wavelength λ = 0.72907 Å. In situ SR-XRD measurements during hydrogen desorption were carried out for Al_0.05_(TiVNb)_0.95_ and TiVNb by applying a constant heating ramp with 2 °C/min from 25 °C up to 450 °C under dynamic secondary vacuum. Quartz capillaries with 1.2 and 1.0 mm diameter were used for Al_0.05_(TiVNb)_0.95_ and TiVNb, respectively. To reduce the X-ray absorbance, the powdered samples were mixed with fumed silica at a mass ratio of 1:2, respectively. The in situ SR-XRD patterns were recorded every 4 °C in the scanning range 10° to 60° (2θ) with the wavelength λ = 0.67156 Å. The phase distribution and related lattice parameters were determined from ex situ and in situ SR-XRD patterns using the Rietveld refinement method implemented in Fullprof software (Thompson-Cox-Hastings pseudo-Voigt function for the peak shape).

The microstructure was analyzed by scanning electron microscope (SEM) using a Zeiss Merlin instrument equipped with an EDX (energy-dispersive X-ray spectroscopy) detector from Oxford Instruments. The powder samples were embedded in an epoxy resin and bulk samples were fixed with MCP 70 alloy, then finely polished and finally coated with a 1.5–2.5 nm Pd layer. For elemental analysis, an accelerated electron voltage of 10 keV was used and the element quantification was performed using Al(K), Ti(K), V(K) and Nb(L) signals. The analyses were carried out for the as-cast and after cycling samples.

The hydrogen desorption properties were analyzed by thermal desorption spectroscopy (TDS) using a home-made instrument with a quadrupole mass spectrometer QMS, as described previously [18]. The procedure consisted of loading about 10 mg of the hydride sample into the sample holder and then connecting it to the quadrupole mass spectrometer working under secondary vacuum (~1 × 10^−6^ mbar). The desorption profile (partial pressures of evolved gases) was recorded by heating the sample at a constant rate of 5 °C/min up to 450 °C. 

## 3. Results and Discussions

### 3.1. Synthesis and Microstructure of the Pristine Alloys

The physicochemical and empirical parameters, such as the lattice distortion (δ, as defined in reference [19]) and the valence electron concentration (VEC), for the Al_x_(TiVNb)_1−x_ alloys with x = 0, 0.05, 0.10, 0.175 and 0.25, are listed in Table 1. For comparison, our previous data for the ternary TiVNb (x = 0) and the quaternary Al_0.10_(TiVNb)_0.90_ (x = 0.10) alloys from reference [17] are also included in the tables, figures and discussion throughout the whole document. 

The lattice distortion (δ) decreased with increase in Al content (Table 1), which was assumed to be related to a decrease in the atom size disparity. The atomic radius of Al is very close to most of the elements (*r*_Ti_ = 1.46 Å > *r*_Nb_ = 1.43 Å > *r*_Al_ = 1.43 Å > *r*_V_ = 1.39 Å [19]), except for V which has the smallest radius. Therefore, increasing Al and decreasing V concentrations led to a decrease in the atom size disparity and, consequently, the lattice distortion reduced. Similar behavior was reported by Ma et al. for the (ZrTiVFe)_x_Al_y_ series, where Al atomic size was very similar to that of the other alloying elements [20]. 

In the same way, the parameter VEC and the bulk density (ρ) were reduced with increasing Al content (Table 1), with the smallest density obtained for the equimolar composition, in agreement with Stepanov et al. [21]. The same lowering of the density by Al substitution to Cr was reported by Senkov et al. for CrMo_0.5_NbTa_0.5_TiZr and AlMo_0.5_NbTa_0.5_TiZr with densities of 8.23 g/cm^3^ and 7.40 g/cm^3^, respectively [22]. 

For all the compositions, δ and VEC were in the range where single-phase *bcc* solid solutions are expected: δ < 6.6% [23] and VEC < 6.87 [24].

**Figure 1:**
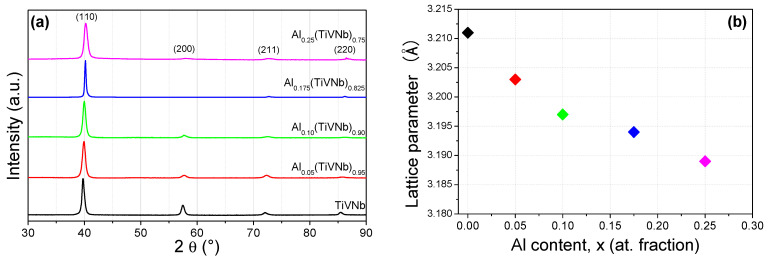
(**a**) XRD patterns of Al_x_(TiVNb)_1−x_ alloys with x = 0 [17], 0.05, 0.10 [17], 0.175, 0.25 (λ = 1.5406 Å). (**b**) *bcc* lattice parameters as a function of Al content.

All as-cast Al_x_(TiVNb)_1−x_ alloys with x = 0 [17], 0.05, 0.10 [17], 0.175 and 0.25 crystallized in a single-phase *bcc* structure, as can be seen in the XRD patterns in Figure 1a. A preferential orientation along the (110) axis was observed with increasing Al content which decreased the relative intensities of the (200) and (211) peaks. The *bcc* lattice parameters, as determined by the Rietveld analysis, are listed in Table 2. Corresponding Rietveld refinements of the Al_0.05_(TiVNb)_0.95_, Al_0.175_(TiVNb)_0.825_ and Al_0.25_(TiVNb)_0.75_ compositions are given in Figure S1 of the Supplementary Information (SI). 

In this series of alloys, only the equimolar composition AlTiVNb has previously been reported by Stepanov et al. [21] and Yurchenko et al. [25]. Both studies described the obtention of a single phase *bcc* lattice with unit cell parameters of 3.18 ± 0.03 Å [21] and 3.186 ± 0.001 Å [25]. These findings are in excellent agreement with the present result (3.189(1) Å for Al_0.25_(TiVNb)_0.75_ in Table 2). 

It is of note that Al has been reported to favor the stabilization of *bcc* phases in HEAs/MPEAs containing refractory metals [26,27] despite its negative enthalpies of mixing with these elements [22]. It was been proposed that the similar atomic size of Al with the alloying refractory elements plays an important role in preventing the formation of intermetallic phases and favors the stabilization of random *bcc* solid solutions [22].

The *bcc* lattice parameters of the series Al_x_(TiVNb)_1−x_ steadily decreased with increasing Al content, as shown in Figure 1b, although the trend was not linear, as also pointed out by Chen et al. in the series of Al_x_NbTiMoV alloys [26]. The reduced lattice parameter with increasing Al content can be explained by decrease in the average atomic size with Al content since its atomic size radius ranges in the intermediate region: *r*_Ti_ = 1.46 Å > *r*_Nb_ = 1.43 Å > *r*_Al_ = 1.43 Å > *r*_V_ = 1.39 Å. Thus, the number of atoms with intermediate sizes increased at the expense of both the largest and the smallest atoms. This behavior has been reported in previous investigations on refractory alloys containing Al [16,17].

**Figure 2:**
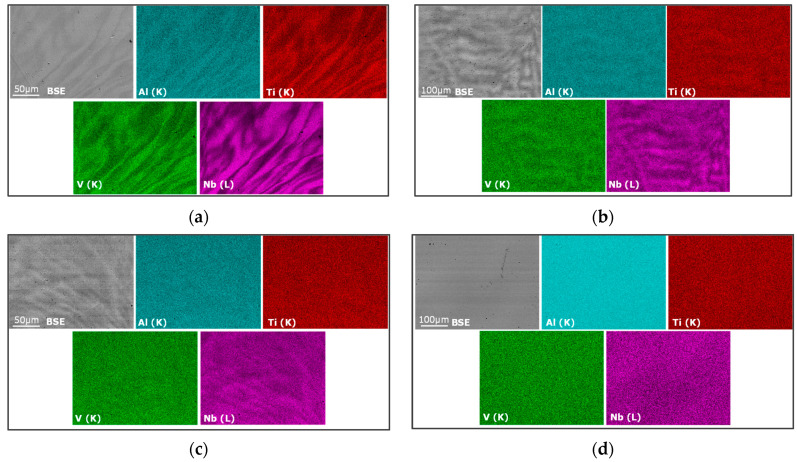
SEM-EDX chemical mapping of the as-cast (**a**) Al_0.05_(TiVNb)_0.95_, (**b**) Al_0.10_(TiVNb)_0.90_, (**c**) Al_0.175_(TiVNb)_0.825_, (**d**) Al_0.25_(TiVNb)_0.75_.

The microstructure and chemical distribution of the as-synthesized compositions with Al are detailed in Figure 2 and Table 3. The overall chemical compositions (Table 3) are in good agreement with the nominal compositions. Dendritic microstructures with a richer Nb composition than interdendritic zones, which are poorer in Nb compared to the nominal concentration, are encountered in all alloys. This microstructure is very common in refractory HEAs [21,26] and has been accounted for by the solidification process—the dendritic zones solidify first and are enriched in elements with the highest melting point, whereas interdendritic regions solidify afterward and contain less high melting temperature metals [28,29]. In our case, Nb had the highest melting point among all the elements (2468 °C_Nb_ > 1902 °C_V_ >1660 °C_Ti_ > 660 °C_Al_), explaining why it was found in the dendritic microstructures. Interestingly, the difference in the Nb concentration between the dendritic and the interdendritric zones tended to lessen with increasing Al content, which suggests that the presence of Al helps to increase the overall chemical homogeneity of these alloys. 

### 3.2. Hydrogen Absorption

**Figure 3:**
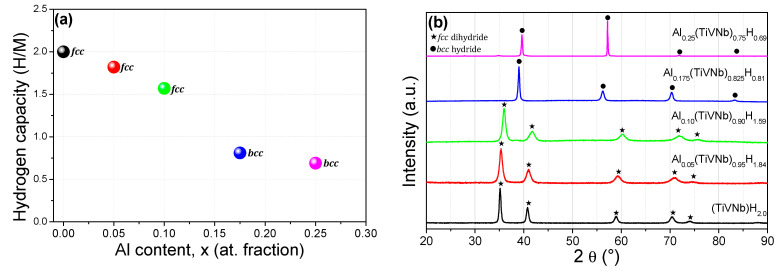
(**a**) Hydrogen storage capacity in terms of H/M as a function of Al content. (**b**) XRD patterns of the Al_x_(TiVNb)_1−x_ hydrides (λ = 1.5406 Å).

First, absorption kinetics were recorded under 38 ± 5 bar H_2_ pressure at 25 °C for the activated Al_x_(TiVNb)_1−x_ alloys with x = 0.05, 0.175 and 0.25. The maximum capacities were: 0.69H/M (1.25 wt.%), 0.81H/M (1.41 wt.%) and 1.84H/M (2.96 wt.%) for Al_0.25_(TiVNb)_0.75_, Al_0.175_(TiVNb)_0.825_ and Al_0.05_(TiVNb)_0.95_, respectively. The capacities obtained under these conditions and the related XRD patterns for the whole series are plotted in Figure 3a,b. A decreasing trend in capacity was observed with increasing Al content, which might be related to the fact that Al does not have a high affinity for hydrogen compared to Ti/V/Nb which are hydride-forming elements [30]. Furthermore, the same fading in capacity with increasing Al content was observed by Ma et al. in (ZrTiVFe)_100−x_Al_x_ [20], which was accounted for by the decrease in lattice distortion with Al addition. 

In terms of gravimetric capacity, the Al_0.05_(TiVNb)_0.95_ alloy has one of the highest reported capacities (2.96 wt.%) among several Al containing alloys, such as, Mg_35_Al_15_Ti_25_V_10_Zn_15_ (2.7 wt.%) [14], Al_0.10_Ti_0.30_V_0.25_Zr_0.10_Nb_0.25_ (2.9 wt.%) [16], (ZrTiVFe)_90_Al_10_ (1.4 wt.%) and (ZrTiVFe)_80_Al_20_ (1.16 wt.%) [20]. The hydrogenated material obtained for the smallest Al amount (x = 0.05) was single-phase *fcc* dihydride (CaF_2_-type, $Fm\bar{3}m$), in agreement with our earlier findings for the initial TiVNb and Al_0.10_(TiVNb)_0.90_ (x = 0.10) [17]. However, for higher Al content (x = 0.175 and 0.25), *bcc* hydrides were observed (Figure 3b). The lattice parameters for the hydride phases are given in Table 2. The Rietveld refinement for *fcc* Al_0.05_(TiVNb)_0.95_H_1.84_ is given in Figure S2. For Al contents x ≥ 0.175, hydrogen absorption induced the formation of *bcc* hydride phases ($Im\bar{3}m$) with slightly increased lattice parameters compared to the as-cast materials due to hydrogen absorption and subsequent lattice expansion (Table 2 and Figure S3). Interestingly, the equimolar composition showed a strong preferential orientation along the (200) direction as indicated by the reinforcement of this diffraction peak relative to the other peak’s intensities (Figure S3b). This might be associated with the occurrence of a well-ordered microstructure following parallel planes induced by hydrogen absorption, as was also observed in an earlier study on TiZrNbTaHf [7]. 

### 3.3. Thermodynamics of Hydrogen Absorption

Hydrogen absorption PCI curves for ternary TiVNb and quaternary Al_0.10_(TiVNb)_0.90_ alloys have previously been reported [17]. Thus, only the PCI curves of Al_0.05_(TiVNb)_0.95_ recorded at several temperatures (25–175 °C) are provided in this section, whereas the discussion of thermodynamics will extend to previous results. 

**Figure 4:**
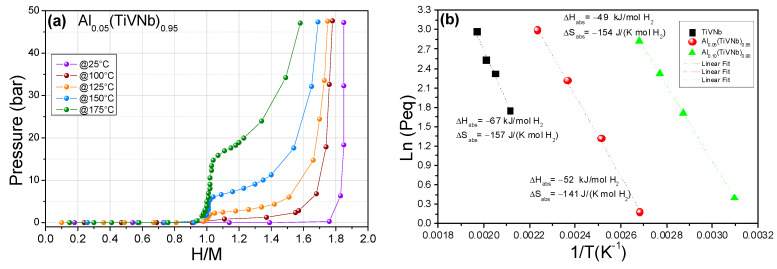
(**a**) PCIs for Al_0.05_(TiVNb)_0.95_ in the temperature range 25–175 °C, (**b**) Van’t Hoff plots for Al_x_(TiVNb)_1−x_ (x = 0 [17], 0.05, 0.10 [17]).

The PCI investigations for Al_0.05_(TiVNb)_0.95_ revealed the existence of two plateaus (Figure 4a): the first plateau occurred at low equilibrium pressure (below the detection limit of the pressure transducer) and ended around 0.9 H/M, irrespective of the temperature. The second plateau arose at high pressure, the value of which increased with temperature. This behavior was in good agreement with the two-step reaction (metal ↔ monohydride ↔ dihydride) already reported for both *bcc* traditional materials [31] and HEAs [7,10,17,32]. The maximum capacity at 25 °C reached 1.84 H/M (2.96 wt.%), in accordance with the full absorption kinetics at room temperature (Section 3.2).

The thermodynamic properties of the dihydride formation in Al_0.05_(TiVNb)_0.95_ have been determined from the Van’t Hoff plots in Figure 4b. For comparison, the Van’t Hoff plots of the ternary TiVNb and quaternary Al_0.10_(TiVNb)_0.90_ are also plotted, based on reference information in [17]. The enthalpies (∆H in kJ/mol H_2_) and entropies (∆S in J/K·mol H_2_) of dihydride formation for x = 0 [17], 0.05 and 0.10 [17] are listed in Table 4 together with the earlier ML predictions [17] with very good agreement observed. 

The increase in Al content in TiVNb led to a progressive decrease in the experimental enthalpy of dihydride formation from −67 to −52 and −49 kJ/mol H_2_ for x = 0, 0.05 and 0.10, respectively. It is of note that a similar thermodynamic destabilization as observed for the addition of x = 0.05 Al content was achieved by the addition of x = 0.25 Cr in Cr_x_(TiVNb)_1−x_ (equimolar composition) [33], suggesting that Al influences hydrogen thermodynamics more effectively than Cr. 

Furthermore, the plateaus of the Al_0.05_(TiVNb)_0.95_ were less sloped than for the ternary pristine alloy, as reported previously [17]. Thus, the decrease in the experimental errors in the thermodynamic calculations with increasing Al content might reasonably be explained by the presence of more flat plateaus in the case of Al-containing alloys. 

**Figure 5:**
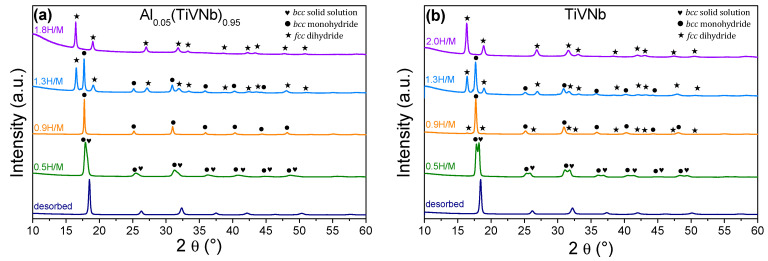
Ex situ SR-XRD patterns (λ = 0.72907 Å) at several stages of the hydrogenation process for (**a**) Al_0.05_(TiVNb)_0.95_ and (**b**) TiVNb.

The two-step transformation induced by hydrogen absorption in Al_0.05_(TiVNb)_0.95_ was investigated by ex situ SR-XRD. The hydrogenation process was stopped at several hydrogen concentrations. The SR-XRD patterns of the intermediate hydrides with uptakes = 0.5, 0.9 and 1.3 H/M, the dihydride (1.8 H/M), as well as the fully desorbed alloy, are displayed in Figure 5a. Furthermore, for comparison, the ex situ SR-XRD results for the intermediate hydrides of the ternary TiVNb are also shown in Figure 5b. The lattice parameters were determined by Rietveld refinements for both compositions (Figures S4 and S5) and are listed in Table 5. 

The partially hydrogenated Al_0.05_(TiVNb)_0.95_H_0.5_ consisted of a mixture of two phases: a *bcc* solid solution with hydrogen with a slightly enlarged lattice parameter (3.255 Å, Table 5) than the initial one (3.203 Å, Table 2) and a *bcc* monohydride. The alloy completely transformed into a *bcc* monohydride at 0.9 H/M with a*_bcc-mh_* = 3.342 Å which was around 4% larger than the initial value (3.203 Å, Table 2). At a hydrogen composition of 1.3 H/M, the material consisted of a mixture of two phases: a *bcc* monohydride (a*_bcc_* = 3.349 Å) and an *fcc* dihydride (a*_fcc_* = 4.392 Å). At full hydrogenation (1.8 H/M) the material was fully transformed into an *fcc* dihydride with a*_fcc_* = 4.412 Å (Table 5). After desorption, the material recovered the *bcc* phase with a very close lattice parameter (3.206 Å, Table 5) compared to the initial alloy (3.203 Å, Table 2). These findings are in good agreement with the PCI curves and indicate that the alloy undergoes a first phase transition from *bcc* to *bcc* monohydride followed by a second transition from *bcc* monohydride to *fcc* dihydride, which correlates well with previous reports on classical *bcc* alloys and more recent *bcc* HEAs. Similar behavior was observed for the pristine TiVNb (Figure 5b and Table 5). 

### 3.4. Hydrogen Desorption

**Figure 6:**
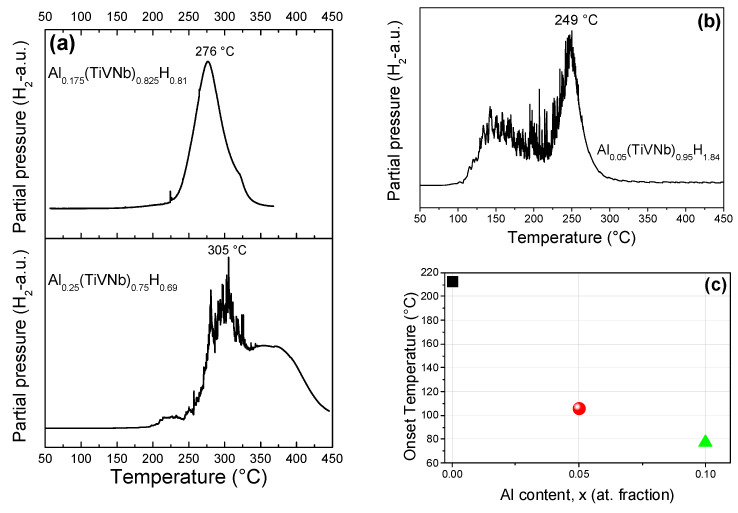
TDS profiles for (**a**) Al_0.175_(TiVNb)_0.825_H_0.81_ (**top**) and Al_0.25_(TiVNb)_0.75_H_0.69_ (**bottom**), (**b**) Al_0.05_(TiVNb)_0.95_H_1.84_, (**c**) variation in the desorption onset temperature as a function of Al content for Al_x_(TiVNb)_1−x_ with x = 0, 0.05 and 0.10.

The desorption properties of the hydrides were determined by thermo-desorption spectroscopy (TDS) with a heating rate of 5 °C/min. The TDS profile of Al_0.175_(TiVNb)_0.825_H_0.81_ showed a main desorption event with a maximum desorption peak at 276 °C, whereas the Al_0.25_(TiVNb)_0.75_H_0.69_ revealed two main desorption peaks with maxima at 305 °C and 375 °C (Figure 6a). 

The TDS profile of Al_0.05_(TiVNb)_0.95_H_1.84_ (Figure 6b) showed two main desorption events with a desorption onset temperature at around 106 °C. The first peak was rather broad with a maximum at around 150 °C, followed by a second sharp peak with a maximum at 249 °C. This two-step profile was in good agreement with our earlier results for Al_0.10_(TiVNb)_0.90_H_1.6_ [17]_,_ though the range of desorption occurred at a lower temperature for the alloy with higher Al content. The desorption onset temperature for the *fcc*-forming hydride materials (x = 0, 0.05 and 0.10) was found to decrease with increasing Al content, as depicted in Figure 6c. Since VEC also depended on the Al content (it decreased with increasing Al content), this trend can be linked with VEC. Thus, the desorption onset temperature increased with the VEC value, as was also recently observed in the series of Ti_0.30_V_0.25_Zr_0.10_Nb_0.25_*X*_0.10_ HEAs [34]. 

The crystalline structure of the desorbed samples was investigated to check their reversibility upon hydrogen absorption and desorption. The XRD patterns and the corresponding Rietveld refinements of the desorbed materials after the TDS measurements (Figure S6) demonstrated that all the desorbed compositions crystallized in a *bcc* lattice with a lattice parameter close to the as-cast material (Table S1). Interestingly, the composition Al_0.25_(TiVNb)_0.75_ after desorption showed a *bcc* structure with a very strong preferential orientation along the (200) direction (Figure S7), which was already introduced by hydrogen absorption (Figure S3b) and retained after desorption.

**Figure 7:**
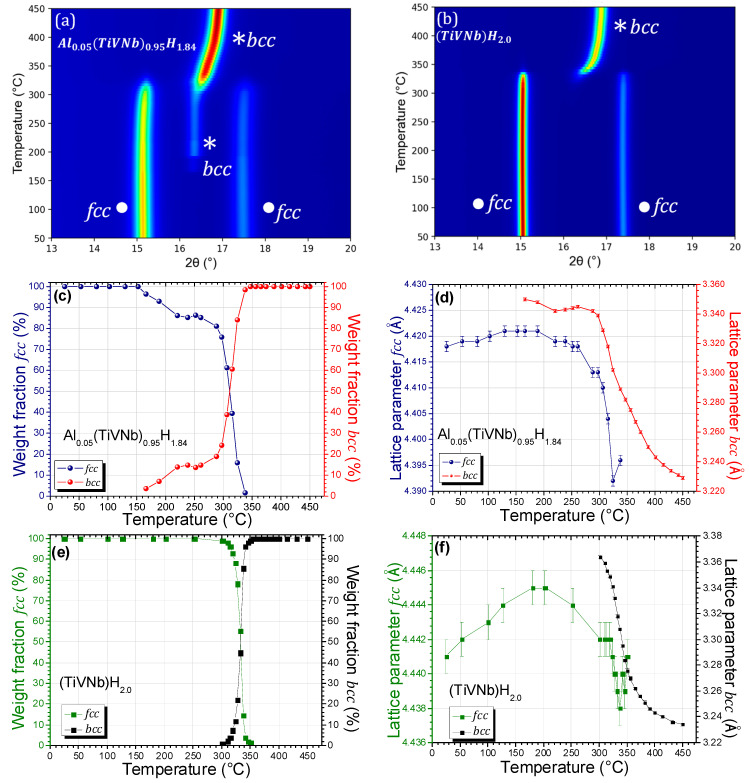
In situ SR-XRD thermo-diffractograms (λ = 0.67156 Å) during hydrogen desorption from (**a**) Al_0.05_(TiVNb)_0.95_H_1.84_ and (**b**) (TiVNb)H_2.0_ under dynamic vacuum from 25 °C up to 450 °C (2 °C/min). Thermal evolution of phase fractions from (**c**) Al_0.05_(TiVNb)_0.95_H_1.84_ and (**e**) (TiVNb)H_2.0_. Thermal evolution of *fcc/bcc* lattice parameters from (**d**) Al_0.05_(TiVNb)_0.95_H_1.84_ and (**f**) (TiVNb)H_2.0_.

To better understand the effect of Al on the desorption process and the related crystalline phases’ evolution, in situ SR-XRD measurements for Al_0.05_(TiVNb)_0.95_H_1.84_ and (TiVNb)H_2.0_ are plotted in Figure 7a,b, respectively. Rietveld refinements were performed to acquire structural information about the variation in phase fraction and lattice parameters during desorption (Figure 7c–f). The full hydrides and desorbed phases of Al_0.05_(TiVNb)_0.95_H_1.84_ and (TiVNb)H_2.0_ are shown in Figure S8a–d, respectively. 

The initial *fcc* dihydride Al_0.05_(TiVNb)_0.95_H_1.84_ (marked with ● in Figure 7a) was stable from 25 °C up to around 150 °C, while the lattice parameter increased from 4.418(1) to 4.421(1) Å (Figure 7d and Table S2) because of the thermal expansion of the crystal lattice. Upon reaching around 160 °C, the *fcc* dihydride started to desorb hydrogen and a new *bcc* phase was formed (marked with * in Figure 7a). This temperature was close to the first desorption event observed in the TDS spectra (Figure 6b), though a shift in temperature could be noticed due to different heating ramps and vacuum environments. At above 160 °C, the two phases coexisted, and the lattice parameter of the newly formed *bcc* phase smoothly decreased, while the parameter for the *fcc* phase ceased to increase and even showed a decrease from 190 °C up to 300 °C (Table S2). This can be explained by the combination of the thermal expansion and the lattice shrinkage by hydrogen desorption occurring at the same time. This coexistence of two phases (*bbc* and *fcc*) has been observed previously by in situ neutron diffraction of Al_0.10_(TiVNb)_0.90_D_1.66_ during deuterium desorption [17]. In the temperature range 250–350 °C the *bcc* phase strongly increased at the expense of the *fcc* dihydride (Figure 7c), while both the *fcc* and *bcc* lattice parameters decreased (Figure 7d and Table S2). This temperature range corresponded to the main sharp TDS event at high temperature (Figure 6b). Starting from 300 °C, the *bcc* lattice parameter began to steadily decrease up to the maximum temperature 450 °C. At 350 °C, the *fcc* phase completely disappeared and the material was transformed into a *bcc* phase. From the thermal evolution of the *bcc* lattice parameter above 300 °C, it seems that this material was still desorbing hydrogen at a maximum temperature of 450 °C. At this temperature, the *bcc* lattice parameter was 3.229(1) Å (Table S2), which was below the value of 3.342 Å obtained for the *bcc* monohydride at room temperature (Table 5). Moreover, the *bcc* lattice parameter value of 3.229(1) Å at 450 °C was larger than 3.206(1) Å obtained for the desorbed phase at room temperature (Table 5). However, this difference can be reasonably explained by the thermal expansion. This suggests that the material had completely desorbed hydrogen under these conditions, and that, at maximum temperature, the transformation from the *fcc* dihydride to the initial *bcc* phase was complete. Thus, from the in situ SR-XRD, only one transition was noticeable from *fcc* to a *bcc* phase without the *bcc* monohydride being discernible under these conditions. 

The in situ SR-XRD profile from the desorption of the ternary (TiVNb)H_2_ (Figure 7b) showed similar behavior, with the main difference being that the *fcc* to *bcc* transition was sharper and occurred in the short temperature range 300–350 °C, without any previous coexistence of the phases. The lattice parameters of *bcc* and *fcc* phases showed a similar trend to that for the Al_0.05_(TiVNb)_0.95_ alloy. Although initially the *fcc* lattice parameter increased with temperature, starting from 200 °C, it decreased, with this phase completely disappearing at around 350 °C. The *bcc* lattice parameter steeply decreased up to 350 °C and approached an almost stable value at higher temperature. Comparable to the Al_0.05_(TiVNb)_0.95_ alloy, the *bcc* lattice parameter at 450 °C was 3.234(1) Å (Table S2), which was smaller than 3.346(1) Å for the *bcc* monohydride at 25 °C (Table 5) but slightly larger than 3.219(1) Å for the desorbed material at room temperature (Table 5). This suggests that the desorption was almost complete for this alloy under the present experimental conditions. However, only one phase transition from *fcc* to *bcc* lattice was observed in the in situ SR-XRD, comparable to the Al_0.05_(TiVNb)_0.95_ alloy.

If both alloys undergo a reversible two-step reaction: *bcc* phase ⇄ *bcc* monohydride ⇄ *fcc* dihydride, as demonstrated by the PCI curves and the ex situ SR-XRD measurement, the question arises: why was the transition from the *bcc* monohydride to the desorbed *bcc* phase not visible from the in situ SR-XRD? Presently, the origin of this effect is not completely certain; however, it might be attributed to the fact that this transition is more difficult to detect in situ as it involves a very small displacement/rearrangement of atoms among the same *bcc* crystal structures, as was previously suggested for Ti_0.30_V_0.25_Cr_0.10_Zr_0.10_Nb_0.25_ [10].

The most important difference between the in situ SR-XRD experiments on Al_0.05_(TiVNb)_0.95_ and the ternary TiVNb was the desorption that occurred at lower temperature for the Al-containing alloy compared to the pristine material. Moreover, both the *fcc* dihydride and the *bcc* desorbed phases coexisted within a large temperature range for Al_0.05_(TiVNb)_0.95_, as was also observed for Al_0.10_(TiVNb)_0.90_ during the in situ neutron diffraction reported in our previous work [17], whereas the ternary alloy showed a sharp transition from the dihydride to the desorbed phase at high temperature. This was in very good agreement with the TDS results and highlights the important role of Al in improving the hydrogen desorption process from these materials. 

### 3.5. Hydrogen Absorption/Desorption Cycling and Kinetics

**Figure 8:**
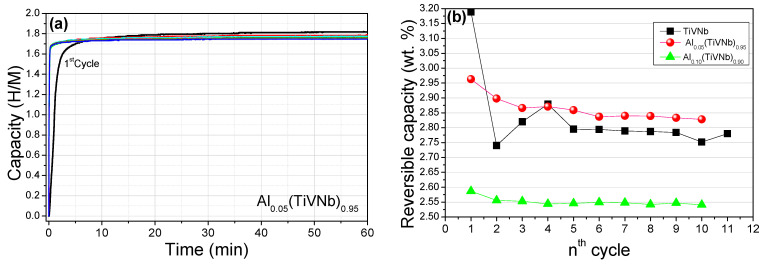
(**a**) Absorption kinetics under final P_eq_ around 32 bar at 25 °C upon 10 cycles for Al_0.05_(TiVNb)_0.95_; the first cycle is in black whereas the subsequent cycles are in color. (**b**) Comparison of the reversible hydrogen absorption capacity (wt.%) upon cycling for Al_x_(TiVNb)_1−x_ (x = 0 [17], 0.05 and 0.10 [17]).

Lastly, Al_0.05_(TiVNb)_0.95_ was submitted to 10 cycles of hydrogen absorption/desorption to evaluate the reversible storage capacity, the crystalline structure stability, and the effect on the reaction kinetics. The cycling process consisted of complete hydrogen absorption with a final P_eq_ around 32 bar at 25 °C, followed by complete desorption at 410 °C under a secondary vacuum for 12 h. The kinetics were recorded during the absorption of hydrogen for each cycle (Figure 8a). The first cycle achieved maximum capacity in less than 40 min and, for subsequent cycles, the absorption kinetics improved by reaching maximum capacity in less than 10 min. This can be accounted for by the hydrogen decrepitation effect that causes the crumbling of alloys and decrease in the grain size. Thus, the kinetics were enhanced by two main processes: (i) new surface areas were created that allowed faster dissociation of hydrogen molecules, and (ii), the diffusion rate within the material increased due to shorter pathways within the grains. 

The capacity slightly decreased from 1.84 H/M to 1.80 H/M after the first cycle and stabilized at 1.76 H/M at the 10th cycle. Interestingly, the stable capacity of Al_0.05_(TiVNb)_0.95_ in terms of wt.% (2.83 wt.%) was higher than both values for Al_0.10_(TiVNb)_0.90_ (2.55 wt.%) and TiVNb (2.8 wt.%) that we reported previously [17], as shown in Figure 8b. The Al_0.05_(TiVNb)_0.95_ alloy lost around 4.5% of the initial capacity until stabilization occurred (from 2.96 wt.% to 2.83 wt.%), which was higher than the 1.8% loss observed for Al_0.10_(TiVNb)_0.90_, but smaller than the 14 % loss for TiVNb. This suggests that Al addition in TiVNb positively affected the reversible capacity when limited Al amounts were used. An optimum capacity was found for x = 0.05 Al content in TiVNb with very good cycling stability and one of the best stable capacities reported so far. The improvement in the stability of the reversible capacity and the gravimetric capacity of hydrogen storage by Al addition as a lightweight element was in very good agreement with the behaviors reported for Ti-V-Zr-Nb with 10 at.% Al [16]. 

After the cycling process, the Al_0.05_(TiVNb)_0.95_H_1.76_ hydride phase was analyzed by XRD (Figure S9b). This demonstrated that the *fcc* dihydride was present along with a monohydride *bcc* phase, suggesting that the absorption was not completed, explaining the observed decrease in capacity during cycling. The same behavior was previously observed for Al_0.10_(TiVNb)_0.90_ and TiVNb [17]. The Rietveld refinements for the cycled phases are given in Figure S9. In addition, the SEM-EDX chemical mapping of Al_0.05_(TiVNb)_0.95_H_1.76_ after 10 cycles is shown in Figure S10. The same microstructure and overall chemical composition (Table S3) as the initial alloy were observed without any phase segregation, in good agreement with earlier results [17]. 

A simple comparison among these three compositions suggests that, not only the addition of non-absorbing lightweight elements in refractory MPEAs is important, but also that the composition must be carefully tuned to optimize both the structure/microstructure of the alloys and their hydrogen sorption performances. 

## 4. Conclusions

The effect of Al addition on the structure, microstructure, and hydrogen sorption properties of a ternary TiVNb alloy was studied from low Al content up to equimolar composition. Al_x_(TiVNb)_1−x_ alloys with x = 0–0.25 were prepared by arc melting as single-phase *bcc* materials with a decreasing lattice parameter by increasing the Al amount. They showed good chemical homogeneity with the typical Nb-rich dendritic microstructure of refractory HEAs. 

The alloys absorbed hydrogen quickly at room temperature with the storage capacity showing a strong dependence on the Al content. Under the experimental conditions used, the richest Al alloys (x = 0.175 and 0.25) formed *bcc* solid solutions after hydrogen absorption with small hydrogen uptakes (0.81 H/M and 0.69 H/M, respectively), whereas the alloys with low Al content (x = 0.05 and 0.10) absorbed large amounts of hydrogen-forming *fcc* hydrides. Moreover, a strong thermodynamic destabilization of the hydride formation was observed with increasing Al content. The enthalpy of hydride formation reduced from −67 kJ/mol H_2_ for the pristine TiVNb alloy to −52 and −49 kJ/mol H_2_ for x = 0.05 and 0.10 Al quantities, respectively. Interestingly, the desorption onset temperatures from the *fcc* dihydride forming materials (x = 0, 0.05 and 0.10 Al) showed a strong decrease with increasing Al content, indicating thermal destabilization of the hydrides by Al addition. 

The Al_0.05_(TiVNb)_0.95_ alloy underwent a reversible two-step reaction from the initial *bcc* to an intermediate *bcc* monohydride and, finally, to an *fcc* dihydride, as confirmed by the two plateaus observed in the PCIs and the ex situ synchrotron XRD. Moreover, a flattening of the plateaus by Al addition was observed which was an important factor in the reduction in errors in the calculation of the thermodynamic properties by the Van’t Hoff equation. Intriguingly, during hydrogen desorption, only one phase transition from the *fcc* dihydride to the *bcc* desorbed state was detectable by in situ SR-XRD for both Al_0.05_(TiVNb)_0.95_H_1.84_ and TiVNb. The expected two-step reaction *fcc* dihydride → *bcc* monohydride → *bcc* desorbed phase was not noticeable, though complete desorption was confirmed by structural analysis. One plausible explanation might be that the *bcc* monohydride → *bcc* desorbed phase transition was more difficult to detect since it involved a very small rearrangement of atoms within the same *bcc* crystalline structure. However, the in situ SR-XRD clearly demonstrated the coexistence of an initial *fcc* dihydride with the *bcc* desorbed phase in a wide temperature range for Al_0.05_(TiVNb)_0.95_, in contrast with the thermal behavior of TiVNb. 

Finally, the Al_0.05_(TiVNb)_0.95_ alloy possessed better cycling properties than both the TiVNb and Al_0.10_(TiVNb)_0.90_ alloys, with a stable reversible capacity of 1.76 H/M (2.83 wt.%), which is one of the best in the recent literature of MPEAs designed for solid-state hydrogen storage.

## Figures and Tables

**Table 1:** Physicochemical (molar mass and bulk density) and empirical parameters (lattice distortion and VEC) of the Al_x_(TiVNb)_1−x_ (x = 0 [17], 0.05, 0.10 [17], 0.175, 0.25) alloys.

Composition	Al Content (at. fraction)	Molar Mass (g/mol)	Density ρ (g/cm^3^)	Lattice Distortion δ (%)	VEC
TiVNb	0	63.9	6.4	4.4	4.7
Al_0.05_(TiVNb)_0.95_	0.05	62.1	6.3	4.3	4.6
Al_0.10_(TiVNb)_0.90_	0.10	60.2	6.1	4.2	4.5
Al_0.175_(TiVNb)_0.825_	0.175	57.4	5.8	4.1	4.4
Al_0.25_(TiVNb)_0.75_	0.25	54.7	5.6	3.9	4.25

**Table 2:** Lattice parameters of the Al_x_(TiVNb)_1−x_ (x = 0 [17], 0.05, 0.10 [17], 0.175, 0.25) series in the form of as-cast alloys and hydrides.

Composition	Form	Phase Structure	Space Group	Lattice Parameter (Å)	Reference
TiVNb	as-cast	*bcc*	*Im-3m*	3.211(2)	[17]
(TiVNb)H_2.0_	dihydride	*fcc*	*Fm-3m*	4.443(1)	
Al_0.05_(TiVNb)_0.95_	as-cast	*bcc*	*Im-3m*	3.203(1)	Present work
Al_0.05_(TiVNb)_0.95_H_1.84_	dihydride	*fcc*	*Fm-3m*	4.418(1)	Present work
Al_0.10_(TiVNb)_0.90_	as-cast	*bcc*	*Im-3m*	3.197(1)	[17]
Al_0.10_(TiVNb)_0.90_H_1.59_	dihydride	*fcc*	*Fm-3m*	4.376(1)	
Al_0.175_(TiVNb)_0.825_	as-cast	*bcc*	*Im-3m*	3.194(2)	Present work
Al_0.175_(TiVNb)_0.825_H_0.81_	hydride	*bcc*	*Im-3m*	3.279(6)	Present work
Al_0.25_(TiVNb)_0.75_	as-cast	*bcc*	*Im-3m*	3.189(1)	Present work
Al_0.25_(TiVNb)_0.75_H_0.69_	hydride	*bcc*	*Im-3m*	3.225(10)	Present work

**Table 3:** EDX chemical analysis of the Al_x_(TiVNb)_1-x_ as-cast alloys with x = 0.05, 0.10, 0.175, 0.25.

Composition	Element	Nominal (at. %)	Overall Average (at. %)	Dendritic Zone (at. %)	Interdendritic Zone (at. %)
Al_0.05_(TiVNb)_0.95_	Al (K)	5	5.1 (0.4)	4.9 (0.2)	5.7 (0.2)
	Ti (K)	31.6	31.4 (1.9)	30.6 (1.3)	34.4 (0.5)
	V (K)	31.6	31.3 (2.0)	30.4 (1.2)	34.6 (0.4)
	Nb (L)	31.6	32.1 (4.2)	34 (2.6)	25.3 (1.1)
Al_0.10_(TiVNb)_0.90_	Al (K)	10	9.8 (0.4)	9.7 (0.4)	10 (0.1)
	Ti (K)	30	29.6 (0.8)	29.3 (0.7)	30.5 (0.3)
	V (K)	30	29.5 (1.1)	28.9 (0.6)	31.1 (0.1)
	Nb (L)	30	31.1 (2.1)	32.1 (1.5)	28.4 (0.5)
Al_0.175_(TiVNb)_0.825_	Al (K)	17.5	16.8 (0.2)	16.7 (0.1)	17 (0.3)
	Ti (K)	27.5	27.5 (0.5)	27.2 (0.5)	28 (0.3)
	V (K)	27.5	27.7 (0.5)	27.4 (0.3)	28.3 (0.1)
	Nb (L)	27.5	28 (1.1)	28.7 (0.5)	26.7 (0.6)
Al_0.25_(TiVNb)_0.75_	Al (K)	25	25.7 (0.8)	25.3 (0.6)	26.5 (0.7)
	Ti (K)	25	24.6 (0.8)	24.2 (0.5)	25.5 (0.8)
	V (K)	25	24.4 (0.3)	24.3 (0.2)	24.8 (0.3)
	Nb (L)	25	25.2 (1.9)	26.2 (1.1)	23.2 (1.6)

**Table 4:** Thermodynamic properties of hydride formation in Al_0.05_(TiVNb)_0.95_ together with already reported experimental and ML predicted values for TiVNb and Al_0.10_(TiVNb)_0.90_ [17].

Composition	ΔH_abs_ (kJ/mol H_2_)		ΔS_abs_ (J/K·mol H_2_)
	Experimental	Machine Learning	
TiVNb	−67 (±5) [17]	−58 [17]	−157 (±11) [17]
Al_0.05_(TiVNb)_0.95_	−52 (±1.5)	−52 [17]	−141 (±3)
Al_0.10_(TiVNb)_0.90_	−49 (±1) [17]	−51 [17]	−154 (±2) [17]

**Table 5:** Lattice parameters of the Al_0.05_(TiVNb)_0.95_ and TiVNb alloys as dihydride (1.8 and 2.0 H/M), partially hydrogenated (0.5 and 1.3 H/M), monohydride (0.9 H/M), and desorbed phases.

Composition	Form	Phase	Phase Fraction (%)	Lattice Parameter (Å)
Al_0.05_(TiVNb)_0.95_H_1.8_	dihydride	*fcc*	100	4.412(1)
Al_0.05_(TiVNb)_0.95_H_1.3_	intermediate	*bcc_mh_* ** fcc	35 65	3.349(1) 4.392(1)
Al_0.05_(TiVNb)_0.95_H_0.9_	monohydride	*bcc_mh_* **	100	3.342(1)
Al_0.05_(TiVNb)_0.95_H_0.5_	intermediate	*bcc_ss_* * bcc_mh_ **	28 72	3.255(1) 3.312(1)
Al_0.05_(TiVNb)_0.95_	desorbed	*bcc*	100	3.206(1)
(TiVNb)H_2.0_	dihydride	*fcc*	100	4.439(1)
(TiVNb)H_1.3_	intermediate	*bcc_mh_* ** fcc	65 35	3.353(1) 4.425(1)
(TiVNb)H_0.9_	monohydride	*bcc_mh_* ** fcc	98 2	3.346(1) 4.423(1)
(TiVNb)H_0.5_	intermediate	*bcc_ss_* * bcc_mh_ **	50 50	3.262(1) 3.328(1)
TiVNb	desorbed	*bcc*	100	3.219(1)

* *bcc_ss_* = *bcc* solid solution. ** *bcc_mh_* = *bcc* monohydride.

## Data Availability

The data used to support the findings of this study are included within the article and the supplementary material.

## Supplementary Materials

The following supporting information can be downloaded at: https://www.mdpi.com/article/10.3390/ma15227974/s1, Figure S1. XRD patterns (λ = 1.5406 Å) and corresponding Rietveld refinement analysis for (a) Al_0.05_(TiVNb)_0.95_, (b) Al_0.175_(TiVNb)_0.825_ and (c) Al_0.25_(TiVNb)_0.75_ Figure S2. Rietveld refinement analysis of the XRD pattern (λ = 1.5406 Å) for the full fcc dihydride Al_0.05_(TiVNb)_0.95_H_1.84_. Figure S3. XRD pattern (λ = 1.5406 Å) for the bcc hydride of (a) Al_0.175_(TiVNb)_0.825_H_0.81_ and (b) Al_0.25_(TiVNb)_0.75_H_0.69_. Figure S4. Rietveld refinement analysis of the ex situ SR-XRD pattern (λ = 0.72907 Å) for the (a) Al_0.05_(TiVNb)_0.95_H_1.8_, (b) Al_0.05_(TiVNb)_0.95_H_1.3_, (c) Al_0.05_(TiVNb)_0.95_H_0.9_, (d) Al_0.05_(TiVNb)_0.95_H_0.5_ and (e) desorbed Al_0.05_(TiVNb)_0.95_. Figure S5. Rietveld refinement analysis of the ex situ SR-XRD pattern (λ = 0.72907 Å) for the (a) (TiVNb)H_2.0_, (b) (TiVNb)H_1.31_, (c) (TiVNb)H_0.9_, (d) (TiVNb)H_0.5_ and (e) desorbed TiVNb. Figure S6. Rietveld refinement analysis of the XRD pattern (λ = 1.5406 Å) after TDS of (a)Al_0.175_(TiVNb)_0.825_, (b)Al_0.10_(TiVNb)_0.90_, (c)Al_0.05_(TiVNb)_0.95_, and (d)TiVNb. Figure S7. XRD pattern (λ = 1.5406 Å) after TDS of (a)Al_0.25_(TiVNb)_0.75_. Figure S8. Rietveld refinement analysis of the in situ SR-XRD pattern during desorption (λ = 0.67156 Å) for the (a) Al_0.05_(TiVNb)_0.95_H_1.84_, (b) desorbed Al_0.05_(TiVNb)_0.95_, (c) (TiVNb)H_2.0_, (d) desorbed TiVNb, everything at 25 °C. Figure S9. Rietveld refinement analysis of the XRD pattern (λ = 1.5406 Å) for the hydride phase after cycling process of (a) Al_0.10_(TiVNb)_0.90_H_1.50_, (b) Al_0.05_(TiVNb)_0.95_H_1.76_, and (c) (TiVNb)H_1.78_. Figure S10. EDX-SEM chemical mapping of the hydride Al_0.05_(TiVNb)_0.95_H_1.76_ after 10 cycles. Table S1. Lattice parameters of the Al_x_(TiVNb)_1−x_ (x = 0 [17], 0.05, 0.10 [17], 0.175, 0.25) desorbed samples after TDS. Table S2. Lattice parameters of the Al_x_(TiVNb)_1−x_ (x = 0, 0.05) alloys from in situ SR-XRD experiments. Table S3. EDX chemical analysis for Al_0.05_(TiVNb)_0.95_H_1.76_ hydride after 10 cycles.
