# Supplementary material for: Uncovering the Effect of Al Addition on the Hydrogen Storage Properties of the Ternary TiVNb Alloy
Source: Materials (Basel). 2022 Nov 11;15(22):7974. doi: 10.3390/ma15227974 (PMC9698924; doi:10.3390/ma15227974)
Supplement: Supplementary file 1 [file materials-15-07974-s001.zip › materials-1985393-supplementary.pdf]

Supplementary information

Uncovering the effect of Al addition on the hydrogen storage properties of the ternary TiVNb alloy

Nayely Pineda Romero[1] and Claudia Zlotea[1]*

[1]Univ. Paris Est Créteil, CNRS, ICMPE, UMR 7182, 2 rue Henri Dunant, 94320, Thiais, France

* Corresponding author: claudia.zlotea@cnrs.fr

Figure SI1. XRD patterns (λ = 1.5406 Å) and corresponding Rietveld refinement analysis for (a) $Al_{0.05}(TiVNb)_{0.95}$, (b) $Al_{0.175}(TiVNb)_{0.825}$ and (c) $Al_{0.25}(TiVNb)_{0.75}$

Figure SI2. Rietveld refinement analysis of the XRD pattern (λ = 1.5406Å) for the full *fcc* dihydride $Al_{0.05}(TiVNb)_{0.95}H_{1.84}$.

Figure SI3. XRD pattern (λ = 1.5406Å) for the *bcc* hydride of (a) $Al_{0.175}(TiVNb)_{0.825}H_{0.81}$ and (b) $Al_{0.25}(TiVNb)_{0.75}H_{0.69}$.

Figure SI4. Rietveld refinement analysis of the *ex situ* SR-XRD pattern (λ= 0.72907Å) for the (a) $Al_{0.05}(TiVNb)_{0.95}H_{1.8}$, (b) $Al_{0.05}(TiVNb)_{0.95}H_{1.3}$, (c) $Al_{0.05}(TiVNb)_{0.95}H_{0.9}$, (d) $Al_{0.05}(TiVNb)_{0.95}H_{0.5}$ and (e) desorbed $Al_{0.05}(TiVNb)_{0.95}$.

Figure SI5. Rietveld refinement analysis of the *ex situ* SR-XRD pattern (λ= 0.72907Å) for the (a) $(TiVNb)H_{2.0}$, (b) $(TiVNb)H_{1.31}$, (c) $(TiVNb)H_{0.9}$, (d) $(TiVNb)H_{0.5}$ and (e) desorbed TiVNb.

Figure SI6. Rietveld refinement analysis of the XRD pattern (λ = 1.5406Å) after TDS of (a)$Al_{0.175}(TiVNb)_{0.825}$, (b)$Al_{0.10}(TiVNb)_{0.90}$, (c)$Al_{0.05}(TiVNb)_{0.95}$, and (d)TiVNb.

Figure SI7. XRD pattern (λ = 1.5406Å) after TDS of (a)$Al_{0.25}(TiVNb)_{0.75}$.

Figure SI8. Rietveld refinement analysis of the *in situ* SR-XRD pattern during desorption (λ= 0.67156Å) for the (a) $Al_{0.05}(TiVNb)_{0.95}H_{1.84}$, (b) desorbed $Al_{0.05}(TiVNb)_{0.95}$, (c) $(TiVNb)H_{2.0}$, (d) desorbed TiVNb, everything at 25°C.

Figure SI9. Rietveld refinement analysis of the XRD pattern (λ = 1.5406Å) for the hydride phase after cycling process of (a) $Al_{0.10}(TiVNb)_{0.90}H_{1.50}$, (b) $Al_{0.05}(TiVNb)_{0.95}H_{1.76}$, and (c) $(TiVNb)H_{1.78}$.

Figure SI10. EDX-SEM chemical mapping of the hydride $Al_{0.05}(TiVNb)_{0.95}H_{1.76}$ after 10 cycles.

Table SI1. Lattice parameters of the $Al_x(TiVNb)_{1-x}$ (x=0 [17], 0.05, 0.10 [17], 0.175, 0.25) desorbed samples after TDS.

Table SI2. Lattice parameters of the $Al_x(TiVNb)_{1-x}$ (x=0, 0.05) alloys from *in situ* SR-XRD experiments.

Table SI3. EDX chemical analysis for $Al_{0.05}(TiVNb)_{0.95}H_{1.76}$ hydride after 10 cycles.

Figure SI1. XRD patterns (λ = 1.5406 Å) and corresponding Rietveld refinement analysis for (a) $Al_{0.05}(TiVNb)_{0.95}$, (b) $Al_{0.175}(TiVNb)_{0.825}$ and (c) $Al_{0.25}(TiVNb)_{0.75}$.

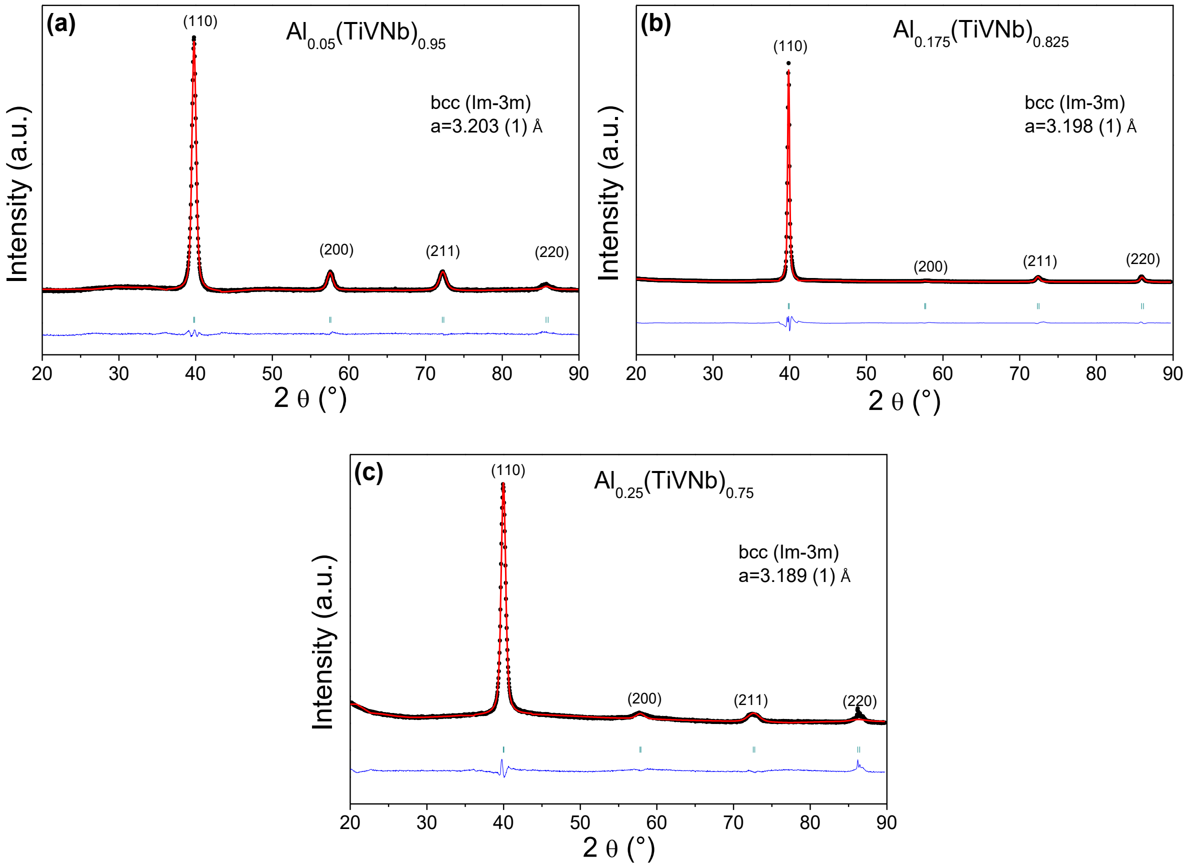

**Figure**	**Composition**	***with all non-excluded points***			**Bragg R-factor**
		**Rp**	**Rwp**	**Chi²**	
SI1a	$Al_{0.05}(TiVNb)_{0.95}$	11.5	6.13	3	1.18
SI1b	$Al_{0.175}(TiVNb)_{0.825}$	15.8	14.5	88	1.78
SI1c	$Al_{0.25}(TiVNb)_{0.75}$	21.9	11.8	11.5	3.47

Figure SI2. Rietveld refinement analysis of the XRD pattern (λ = 1.5406Å) for the full *fcc* dihydride $Al_{0.05}(TiVNb)_{0.95}H_{1.84}$.

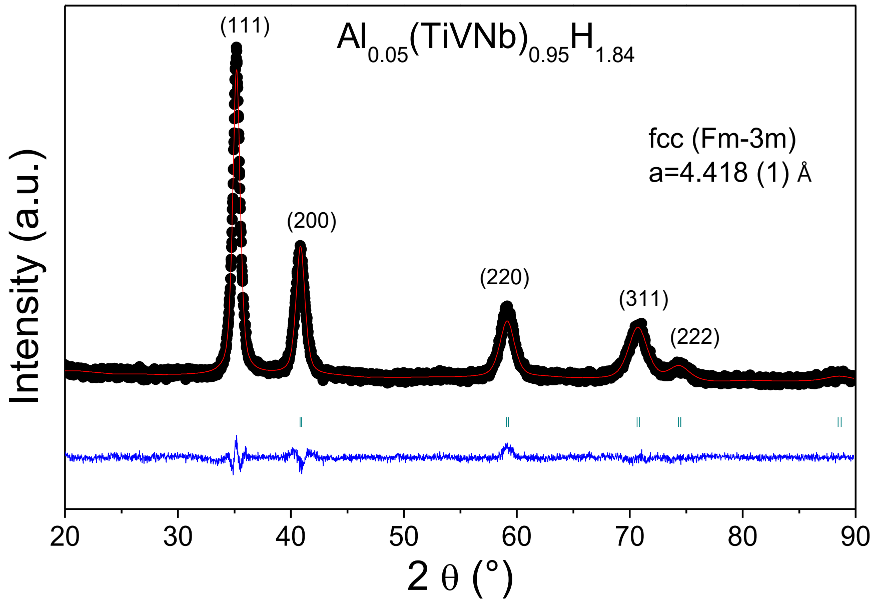

Figure	Composition	*with all non-excluded points*			Bragg R-factor
		Rp	Rwp	Chi²	
SI2	$Al_{0.05}(TiVNb)_{0.95}H_{1.84}$	18.2	15.1	1.52	2.01

Figure SI3. XRD patter (λ = 1.5406Å) for the *bcc* hydride of (a) $Al_{0.175}(TiVNb)_{0.825}H_{0.81}$ and (b) $Al_{0.25}(TiVNb)_{0.75}H_{0.69}$.

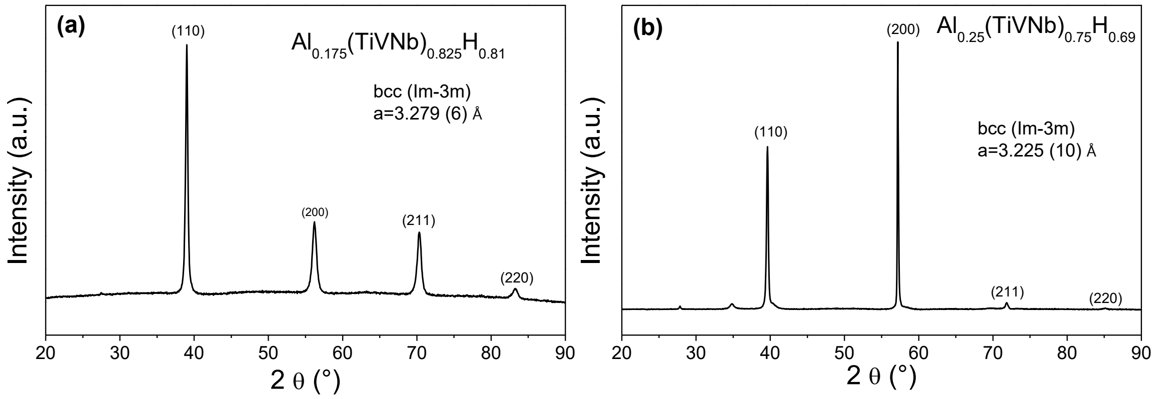

Lattice parameter obtained by CELREF unit cell refinement of powder diffraction data software (http://ccp14.cryst.bbk.ac.uk/soluti on/unitcellrefine/index.html).

Figure SI4. Rietveld refinement analysis of the *ex situ* SR-XRD pattern (λ= 0.72907Å) for the (a) $Al_{0.05}(TiVNb)_{0.95}H_{1.8}$, (b) $Al_{0.05}(TiVNb)_{0.95}H_{1.3}$, (c) $Al_{0.05}(TiVNb)_{0.95}H_{0.9}$, (d) $Al_{0.05}(TiVNb)_{0.95}H_{0.5}$ and (e) desorbed $Al_{0.05}(TiVNb)_{0.95}$.

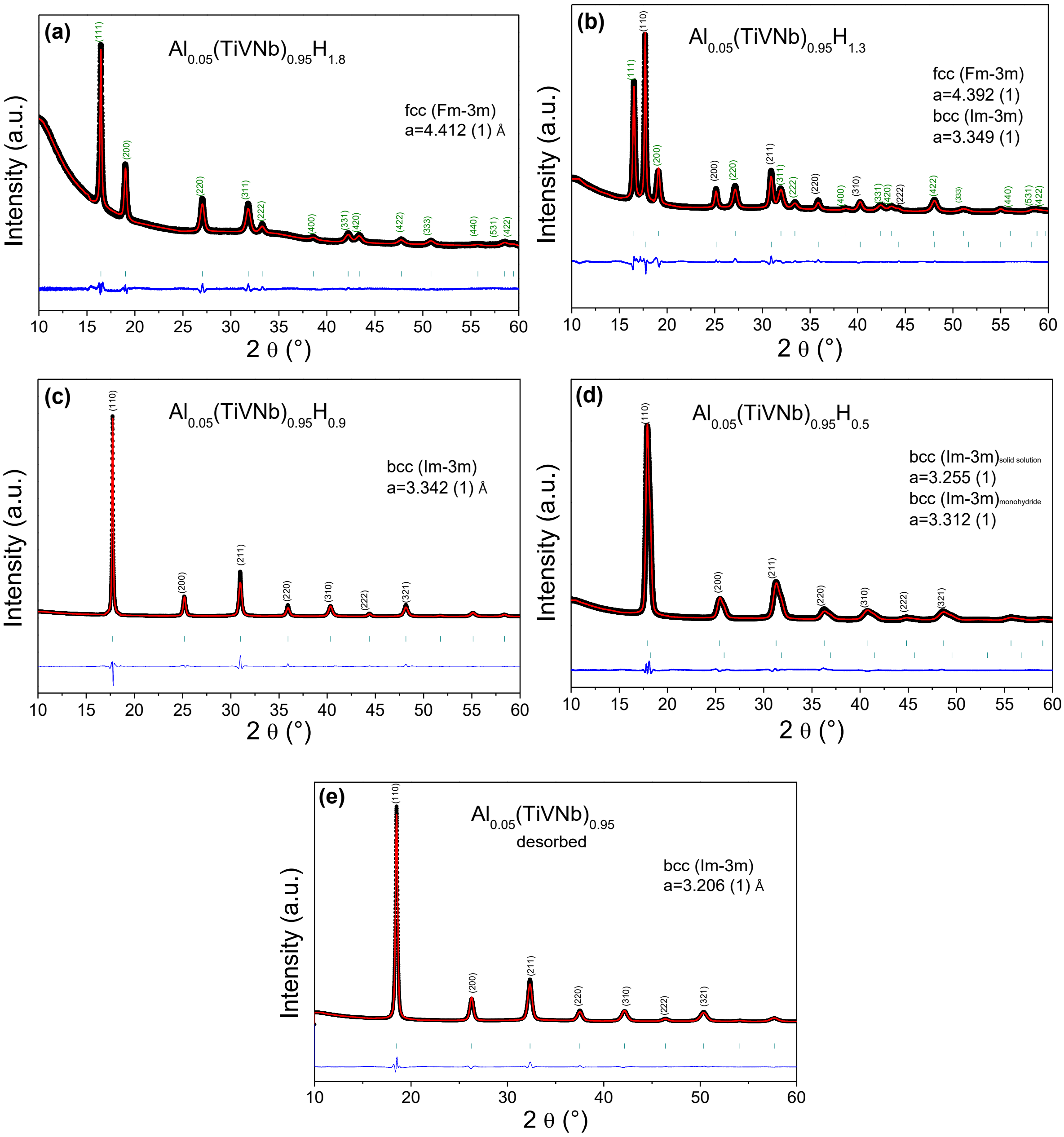

Figure	Composition	*with all non-excluded points*			Bragg R-factor
		Rp	Rwp	Chi²	
SI4a	$Al_{0.05}(TiVNb)_{0.95}H_{1.8}$	25.4	15.9	3.47	6.94
SI4b	$Al_{0.05}(TiVNb)_{0.95}H_{1.3}$	11.8	12.0	12.9	3.40 fcc<br>3.48 bcc
SI4c	$Al_{0.05}(TiVNb)_{0.95}H_{0.9}$	12.0	15.0	33.7	4.45
SI4d	$Al_{0.05}(TiVNb)_{0.95}H_{0.5}$	8.83	10.8	14.9	4.51 bcc monohydride<br>5.42 bcc solid solution
SI4e	$Al_{0.05}(TiVNb)_{0.95}$ desorbed	8.66	10.6	16.3	1.53

Figure SI5. Rietveld refinement analysis of the *ex situ* SR-XRD pattern (λ= 0.72907Å) for the (a) $(TiVNb)H_{2.0}$, (b) $(TiVNb)H_{1.31}$, (c) $(TiVNb)H_{0.9}$, (d) $(TiVNb)H_{0.5}$ and (e) desorbed TiVNb.

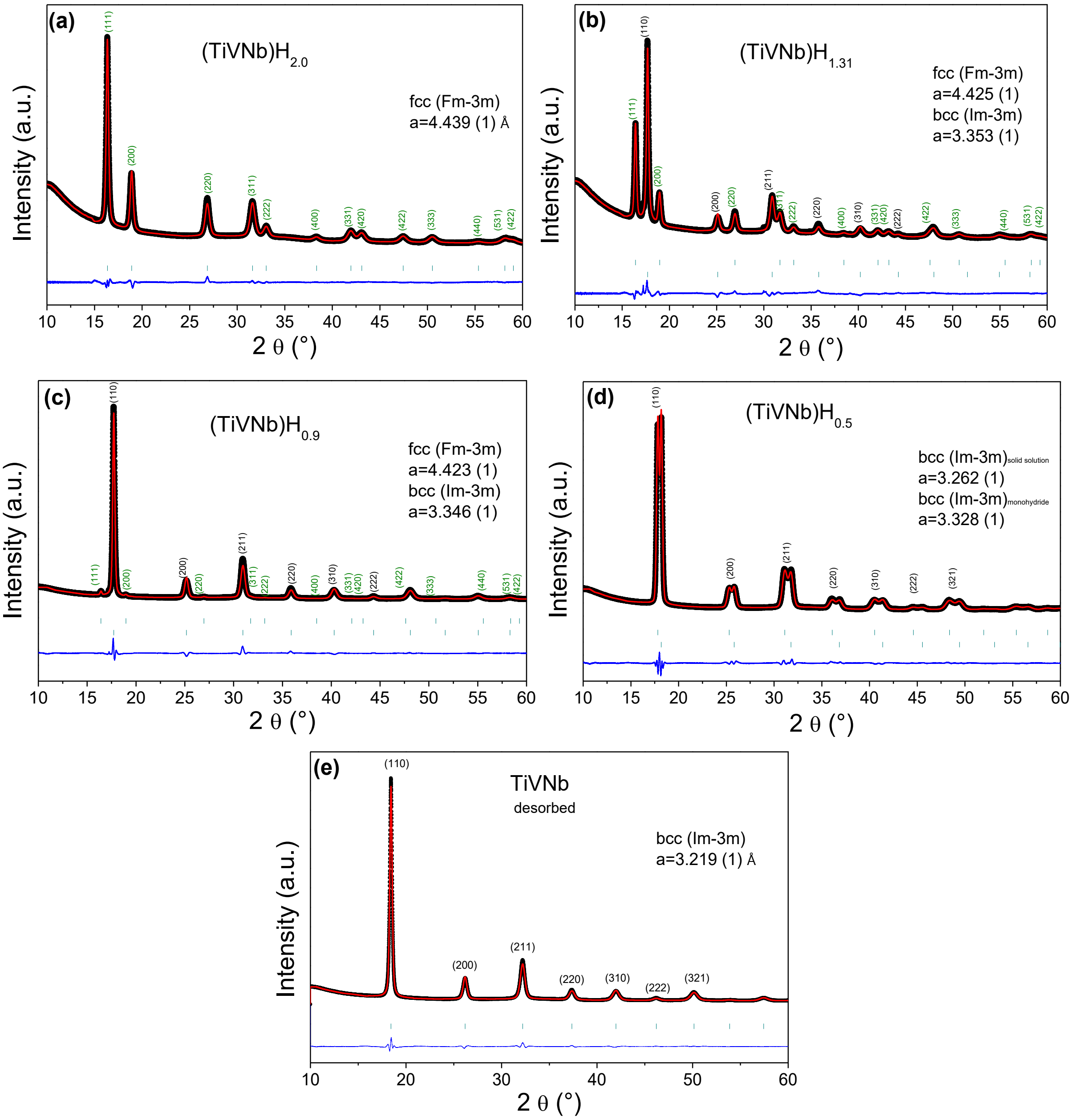

Figure	Composition	with all non-excluded points			Bragg R-factor
		Rp	Rwp	Chi^2	
SI5a	$(TiVNb)H_{2.0}$	8.96	8.18	3.94	3.22
SI5b	$(TiVNb)H_{1.31}$	12.5	12.9	9.87	4.66 fcc<br>6.50 bcc
SI5c	$(TiVNb)H_{0.9}$	12.4	14.2	24.1	31.6 fcc<br>4.91 bcc
SI5d	$(TiVNb)H_{0.5}$	7.24	8.08	9.15	2.67 bcc monohydride<br>2.94 bcc solid solution
SI5e	TiVNb desorbed	7.91	9.64	11.2	2.05

Figure SI6. Rietveld refinement analysis of the XRD pattern (λ = 1.5406Å) after TDS of (a) $Al_{0.175}(TiVNb)_{0.825}$, (b) $Al_{0.10}(TiVNb)_{0.90}$, (c) $Al_{0.05}(TiVNb)_{0.95}$, and (d) TiVNb.

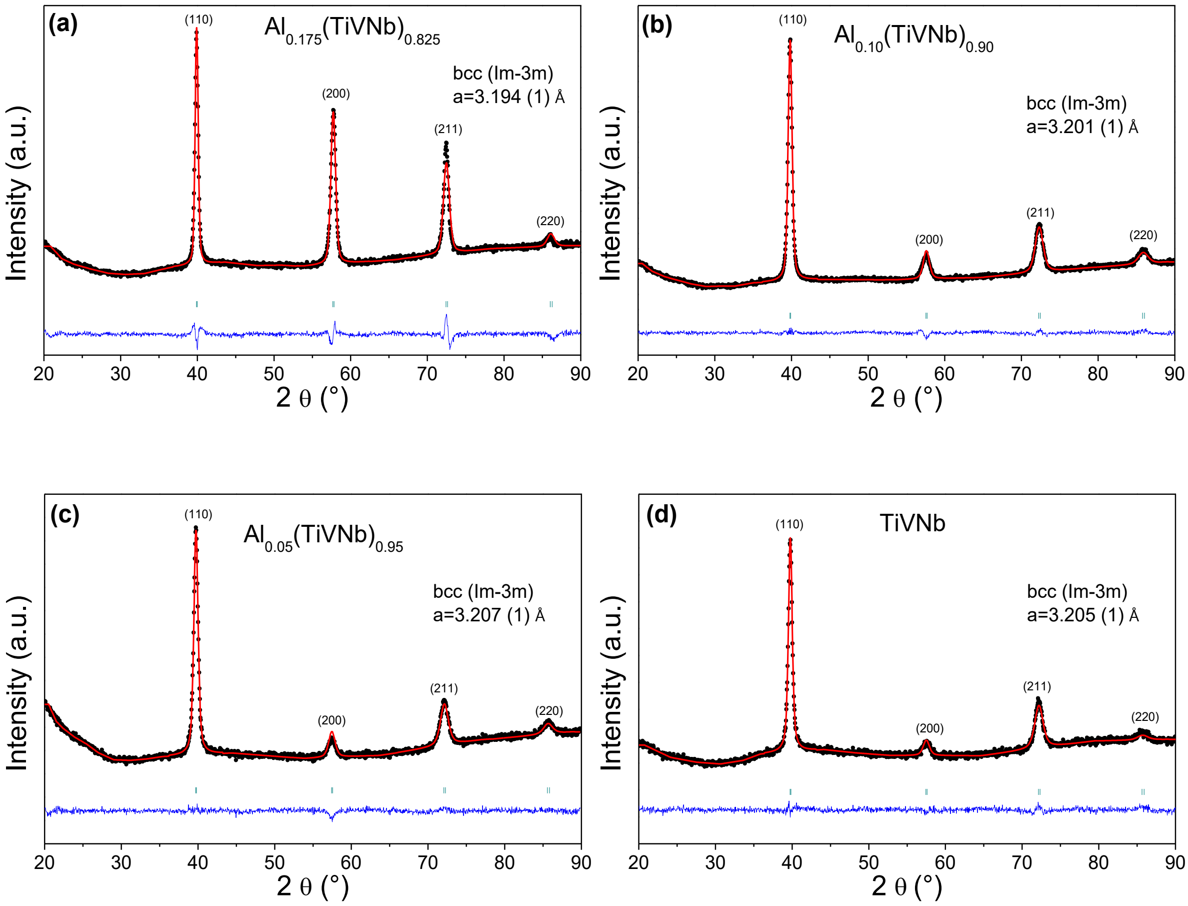

Figure	Composition	*with all non-excluded points*			Bragg R-factor
		Rp	Rwp	Chi²	
SI6a	$Al_{0.175}(TiVNb)_{0.825}$	26.5	13.9	2.67	1.98
SI6b	$Al_{0.10}(TiVNb)_{0.90}$	21.8	10.4	1.50	1.87
SI6c	$Al_{0.05}(TiVNb)_{0.95}$	31.3	13.8	1.44	1.53
SI6d	TiVNb	37.1	15.1	1.28	2.38

Figure SI7. XRD pattern (λ = 1.5406Å) after TDS of (a)$Al_{0.25}(TiVNb)_{0.75}$.

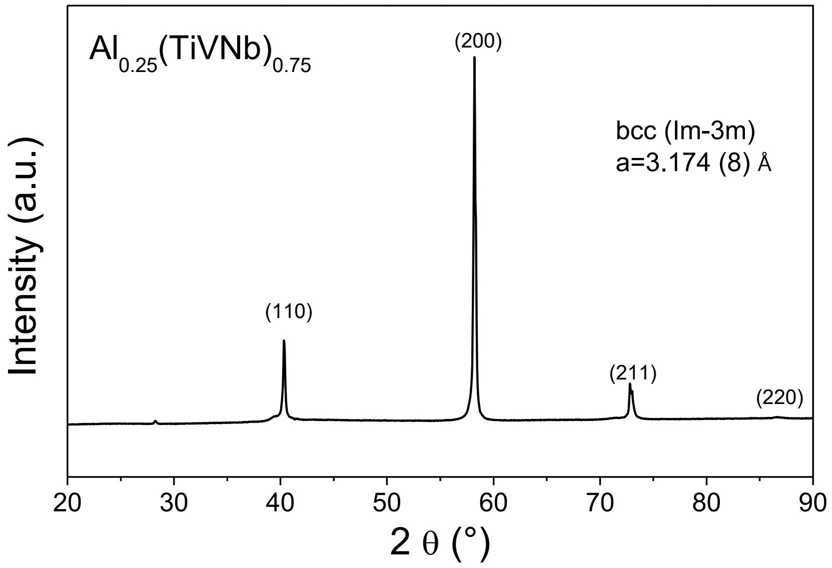

Figure SI8. Rietveld refinement analysis of the *in situ* SR-XRD pattern during desorption (λ= 0.67156Å) for the (a) $Al_{0.05}(TiVNb)_{0.95}H_{1.84}$, (b) desorbed $Al_{0.05}(TiVNb)_{0.95}$, (c) $(TiVNb)H_{2.0}$, (d) desorbed TiVNb at 25°C.

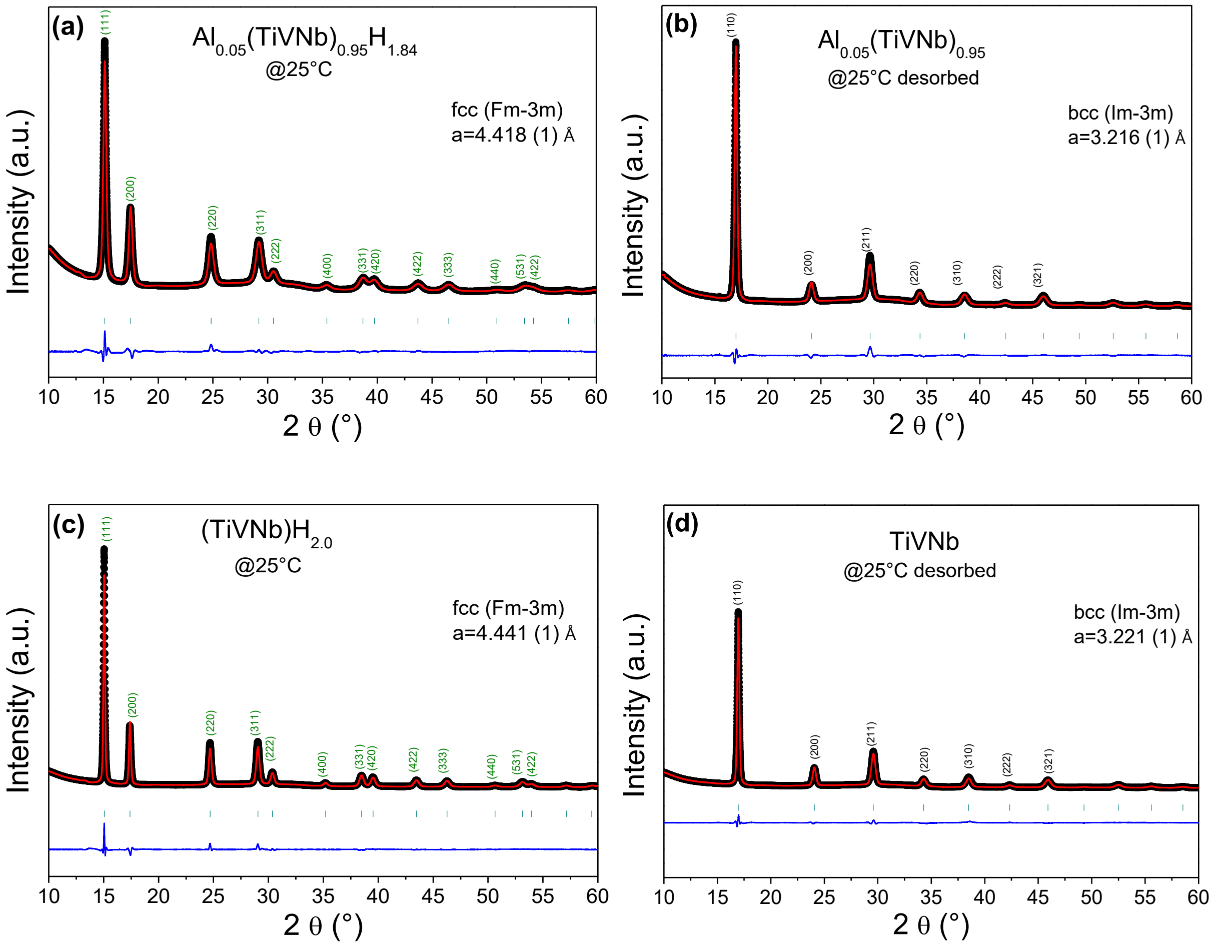

Figure	Composition	*with all non-excluded points*			Bragg R-factor
		Rp	Rwp	Chi^2	
SI8a	$Al_{0.05}(TiVNb)_{0.95}H_{1.84}$	9.73	10.6	15.6	3.69
SI8b	$Al_{0.05}(TiVNb)_{0.95}$	10.5	12.2	13.9	4.18
SI8c	$(TiVNb)H_{2.0}$	10.6	11.8	21.8	5.79
SI8d	TiVNb	9.07	11.0	13.2	4.46

Figure SI9. Rietveld refinement analysis of the XRD pattern (λ = 1.5406Å) for the hydride phase after cycling process of (a) $Al_{0.10}(TiVNb)_{0.90}H_{1.50}$, (b) $Al_{0.05}(TiVNb)_{0.95}H_{1.76}$, and (c) $(TiVNb)H_{1.78}$.

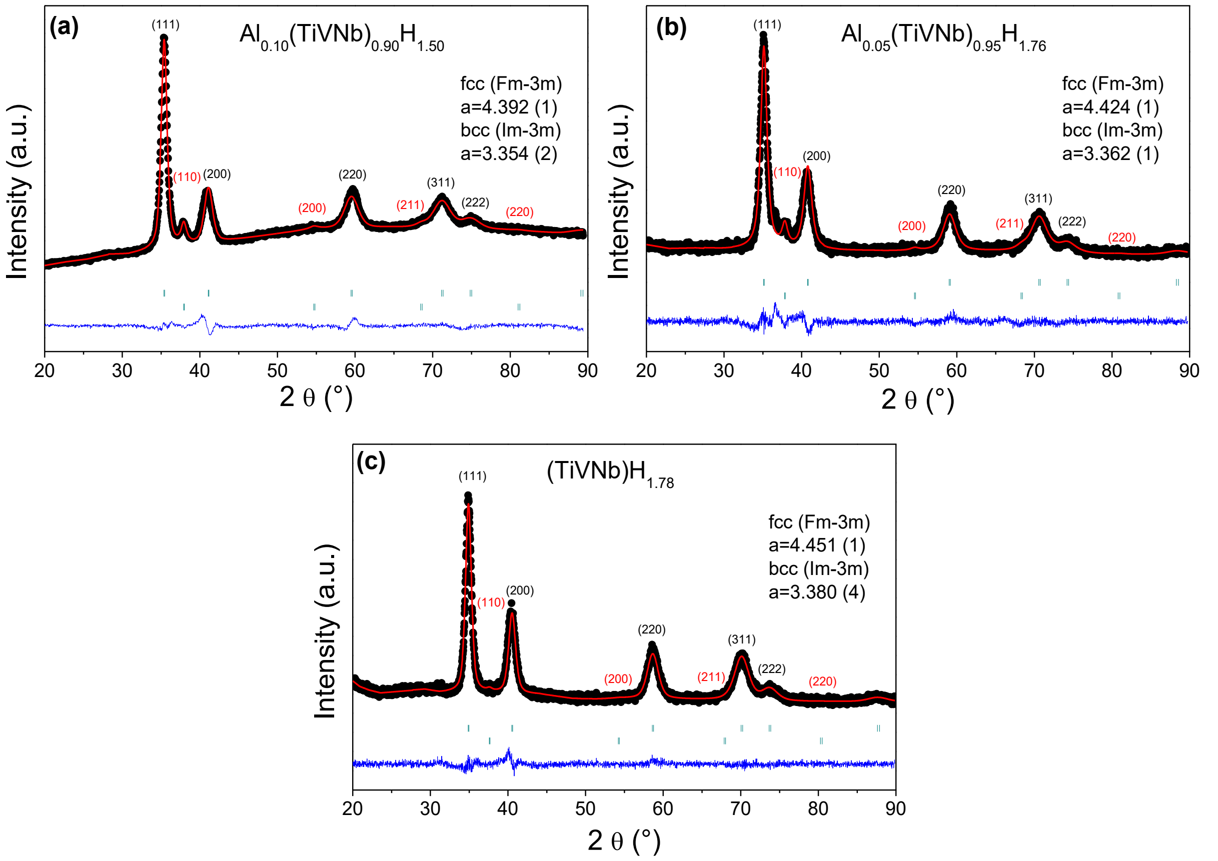

Figure	Composition	with all non-excluded points			Bragg R-factor
		Rp	Rwp	Chi²	
SI9a	$Al_{0.10}(TiVNb)_{0.90}H_{1.50}$	16.3	12.1	3.40	2.53 fcc<br>6.98 bcc
SI9b	$Al_{0.05}(TiVNb)_{0.95}H_{1.76}$	20.9	18.1	1.84	1.93 fcc<br>9.00 bcc
SI9c	$TiVNbH_{1.78}$	21.1	15.9	1.35	2.97 fcc<br>6.59 bcc

Figure SI10. EDX-SEM chemical mapping of the hydride $Al_{0.05}(TiVNb)_{0.95}H_{1.76}$ after 10 cycles.

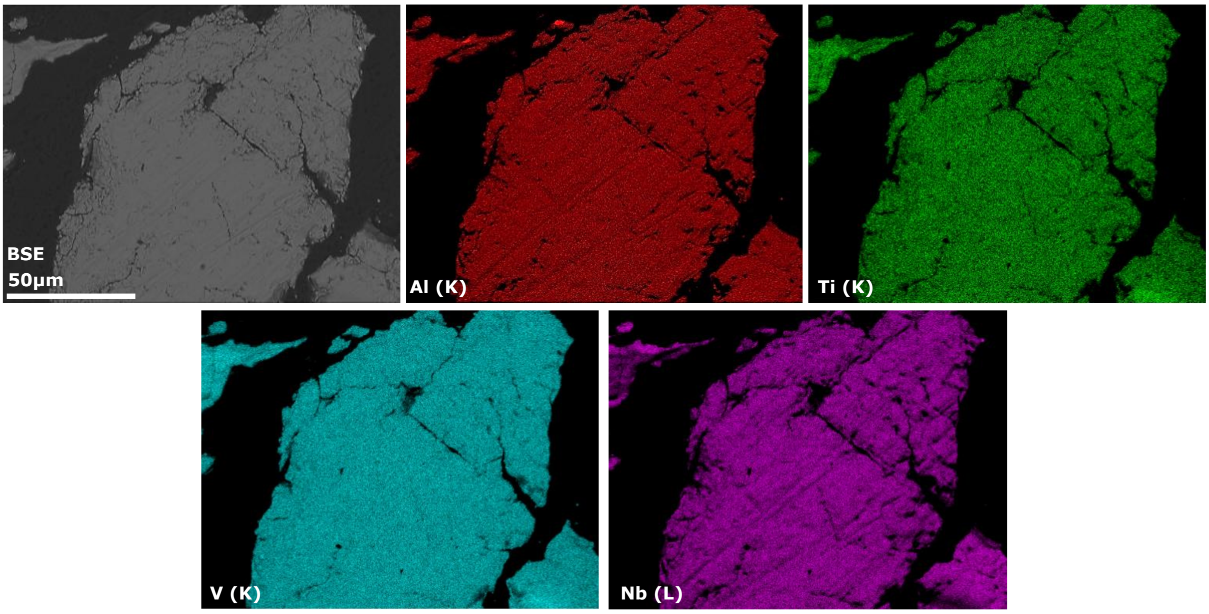

Table SI1. Lattice parameters of the $Al_x(TiVNb)_{1-x}$ (x=0 [17], 0.05, 0.10 [17], 0.175, 0.25) desorbed samples after TDS.

Composition	Phase structure	Lattice parameter (Å)
$Al_{0.25}(TiVNb)_{0.75}$	*bcc*	3.174 (8) *
$Al_{0.175}(TiVNb)_{0.825}$	*bcc*	3.194(1)
$Al_{0.10}(TiVNb)_{0.90}$	*bcc*	3.201(1)
$Al_{0.05}(TiVNb)_{0.95}$	*bcc*	3.207(1)
TiVNb	*bcc*	3.205(1)

*Obtained by CELREF.

Table SI2. Lattice parameters of the $Al_x(TiVNb)_{1-x}$ (x=0, 0.05) alloys from *in situ* SR-XRD experiments.

Composition	Form	Phase structure	Phase fraction (%)	Lattice parameter (Å)	Temperature (°C)
$Al_{0.05}(TiVNb)_{0.95}H_{1.84}$	dihydride	*fcc*	100	4.418(1)	25
$Al_{0.05}(TiVNb)_{0.95}$		*fcc*	100	4.421 (1)	152.3
		bcc			
		bcc_{mh}**	3.59	3.350 (1)	166
		fcc	96.41	4.421 (1)	
		bcc_{mh}**	7.03	3.348 (1)	188.7
		fcc	92.97	4.421 (1)	
		bcc_{mh}**	13.70	3.344 (1)	252
		fcc	86.30	4.418(1)	
		bcc_{mh}**	60.57	3.318(1)	315.4
		fcc	39.43	4.404(1)	
		bcc	100	3.282(1)	347
		bcc	100	3.229(1)	450.1
$Al_{0.05}(TiVNb)_{0.95}$	desorbed	*bcc*	100	3.216(1)	25

$(TiVNb)H_{2.0}$	dihydride	*fcc*	100	4.441(1)	25
TiVNb		*fcc*	100	4.445(1)	180.1
		fcc	100	4.445 (1)	251.9
		bcc_{mh}** *fcc*	0.83 99.17	3.364(1) 4.442(1)	301.2
		bcc_{mh}** *fcc*	44.73 55.27	3.318(1) 4.439(1)	332.7
		bcc_{mh}** *fcc*	85.67 14.33	3.308(1) 4.438(1)	337.2
		bcc	100	3.270(1)	355.1
		bcc	100	3.234(1)	449.6
TiVNb	desorbed	*bcc*	100	3.221(1)	25

* bcc_{ss}= *bcc solid solution* ** bcc_{mh}=*bcc monohydride*

Table SI3. EDX chemical analysis for $Al_{0.05}(TiVNb)_{0.95}H_{1.76}$ hydride after 10 cycles.

Composition	Element	Nominal (at. %)	Overall average (at. %)	Nb rich region (at. %)	Nb poor region (at. %)
$Al_{0.05}(TiVNb)_{0.95}$	Al (K)	5	4.61 (0.22)	4.63 (0.22)	4.62 (0.21)
	Ti (K)	31.6	31.58 (0.93)	31.11(0.77)	32.38(0.66)
	V (K)	31.6	31.48 (0.97)	30.96(0.79)	32.44(0.51)
	Nb (L)	31.6	32.32 (1.74)	33.30 (1.28)	30.55(1.08)
